# A novel recombinant pseudorabies virus expressing parvovirus VP2 gene: Immunogenicity and protective efficacy in swine

**DOI:** 10.1186/1743-422X-8-307

**Published:** 2011-06-16

**Authors:** Yang Chen, Wanzhu Guo, Zhiwen Xu, Qigui Yan, Yan Luo, Qian Shi, Dishi Chen, Ling Zhu, Xiaoyu Wang

**Affiliations:** 1 Animal Biotechnology Center of Sichuan Agricultural University, Ya'an, Sichuan, 625014, PR China; 2 Sichuan Agricultural University Dujiangyan Campus, Dujiangyan, Sichuan, 611830, PR China; 3 Center of Epizootic Prevention & Supervision of Sichuan, Chengdu, Sichuan, 610041, PR China

**Keywords:** Recombinant pseudorabies virus, Porcine parvovirus, VP2 gene, Immunogenicity, Protective Efficacy

## Abstract

**Background:**

Porcine parvovirus (PPV) VP2 gene has been successfully expressed in many expression systems resulting in self-assembly of virus-like particles (VLPs) with similar morphology to the native capsid. Here, a pseudorabies virus (PRV) system was adopted to express the PPV VP2 gene.

**Methods:**

A recombinant PRV SA215/VP2 was obtained by homologous recombination between the vector PRV viral DNA and a transfer plasmid. Then recombinant virus was purified with plaque purification, and its identity confirmed by PCR amplification, Western blot and indirect immunofluorescence (IFA) analyses. Electronic microscopy of PRV SA215/VP2 confirmed self-assembly of both pseudorabies virus and VLPs from VP2 protein.

**Results:**

Immunization of piglets with recombinant virus elicited PRV-specific and PPV-specific humoral immune responses and provided complete protection against a lethal dose of PRV challenges. Gilts immunized with recombinant viruses induced PPV-specific antibodies, and significantly reduced the mortality rate of (1 of 28) following virulent PPV challenge compared with the control (7 of 31). Furthermore, PPV virus DNA was not detected in the fetuses of recombinant virus immunized gilts.

**Conclusions:**

In this study, a recombinant PRV SA215/VP2 virus expressing PPV VP2 protein was constructed using PRV SA215 vector. The safety, immunogenicity, and protective efficacy of the recombinant virus were demonstrated in piglets and primiparous gilts. This recombinant PRV SA215/VP2 represents a suitable candidate for the development of a bivalent vaccine against both PRV and PPV infection.

## Background

Porcine parvovirus (PPV) is a major cause of the syndrome of reproductive failure observed in sows. The infection occurs without clinical symptoms in adults. However, the virus crosses the placental barrier, infecting embryos and leading to stillbirths. Recent studies have indicated that, in addition to inducing reproductive failure, PPV also causes dermatitis, diarrhea, and respiratory system disease [1-3]. A program of continuous vaccination is required to avoid the substantial economic losses associated with this globally prevalent virus.

Parvovirus infections are controlled mainly by the humoral immune responses [4], Therefore, it is hypothesized that generation of effective immunity in sows prior to conception will prevent PPV infection [5]. It is generally believed that active acquired immunity against PPV provides lifelong protection against clinical diseases. Classical vaccines based on inactivated viruses are still in use [6]. However, safety considerations together with poor PPV replication in vitro have intensified the need to develop alternative vaccines.

The major structural protein, VP2 is the main target for neutralizing antibodies in PPV. Many systems have been used to express VP2 resulting in successful self-assembly of virus-like particles (VLPs) with similar morphology to the native capsid and identical hemagglutination activity compared with active PPV. The PPV VLPs have been extensively studied due to their ability to induce a whole range of immune responses [7]. The application of this technology is expected to be important in the development of novel vaccines for PPV.

Live vaccines based on recombinant viruses have played an important role in the development of new vaccines. Live attenuated pseudorabies virus (PRV) has been proven as an excellent vector for expression and delivery of heterologous antigens in the development of recombinant vaccines against infectious diseases in swine [8-15] due to a number of advantageous characteristics. The genome structure and genetic background are relatively well defined and multiplication and stable expression of foreign genes does not affect the stability of the virus itself. Furthermore, the virus infects a wide range of hosts which do not jeopardize human safety. The large DNA genome of PRV is capable of accommodating several kilobases (kb) of foreign DNA and a number of appropriate insertion sites and useful promoters have been identified [16]. These insertion sites include TK, PK, gE, gI, and gG genes, all of which are nonessential for viral replication [16,17]. Inactivation or deletion of one or more of these genes leads to an attenuated phenotype while retaining the replication ability of the virus [18]. Based on the attenuated live vaccine, several PRV recombinants expressing immunogens of heterologous pathogens, such as the glycoprotein E1 of classical swine fever virus (CSFV) have been constructed and vaccination with the recombinant PRV has been shown to confer protection against Aujeszky's disease and classical swine fever. These observations have clearly demonstrated the significance of attenuated PRV in development of bi- or multi-valent vaccines to control animal diseases [15].

In this report, immunogenicity and protective efficacy of a recombinant pseudorabies virus expressing PPV VP2 protein was demonstrated in swine with the aim of providing a novel vaccine to be used in prevention and control of both PRV and PPV infections in the future.

## Materials and methods

### Virus, cells and plasmid

The parent virus PRV SA215, which has been widely used in China to control Aujeszky's disease, is a PRV Fa derivative in which genes encoding three important virulence factors (TK, gE, and gI) have been deleted. Generation of the construct has been described in our laboratory previously [19,20]. The PPV-SC1 strain was isolated from the stillbirth of field pigs in Sichuan province, China. Vero cells and ST cells were cultured in Dulbecco's modified Eagle's medium (DMEM, Gibco, USA), supplemented with 10% (v/v) fetal calf serum (FCS, Gibco, USA) , 100 IU/ml of streptomycin, and 100 IU/ml of penicillin at 37°C in a humidified 5% CO_2 _atmosphere. The transfer plasmid pPI-2.EGFP containing PRV homology region and enhanced green fluorescent protein (EGFP) gene was constructed in this laboratory.

### Construction of recombinant transfer plasmids

PPV viral DNA was extracted from the ST cells infected by the PPV-SC1 strain and used as a template to amplify the PPV VP2 gene using following pair of specific primers, Forward: 5'-tta ggt acc atg agt gaa aat gtg gaa c -3', Reverse: 5'-ttt gga tcc gta taa ttt tct tgg tat aag-3'. Restriction enzyme sites (*Kpn *I and *Bam*H I, indicated by underlining) were introduced into the forward and reverse primers respectively for cloning purposes.The 1740-bp PCR product was digested by *Kpn *I and *Bam*H I, and inserted into the corresponding sites in the PRV transfer plasmid pPI-2.EGFP. The resulting construct encoded EGFP at the N-terminus of VP2 gene and was designated as pPI-2.EGFP.VP2. Identity of the construct was confirmed by diagnostic restriction enzyme digestion.

### Generation of recombinant PRV SA215/VP2 virus

**Figure 1:**
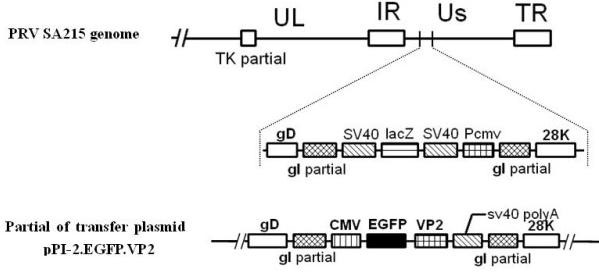
**Construction of recombinant PRV SA215/VP2**. The PRV SA215 genomic DNA was approximately 150 kb including a unique long region (UL), a unique short region (US), internal repeat (IR) and terminal repeat (TR). The thymidine kinase gene (TK) was partially deleted. PRV SA215/VP2 was generated from PRV SA215/VP2. The LacZ expression cassette was replaced with the EGFP/VP2 expression cassette by homologous recombination.

Vero cells were co-transfected with plasmid pPI-2.EGFP.VP2 and genomic DNA of PRV SA215 using lipofectamine TM 2000 reagent (Invitrogen, USA) according to the instructions provided by the manufacturer. Infected cells containing recombinant pseudorabies virus (PRV SA215/VP2) were harvested when obvious cytopathic effect (CPE) was observed (Figure 1). The recombinant viruses were purified five times by plaque-formation assay. In each round, green plaques identified by fluorescent microscopy were harvested, and the presence of VP2 gene was confirmed by PCR amplifying.

### Western blot and indirect immunofluorescence assay

VP2 protein expression in the recombinant PRV SA215/VP2 was detected by Western blot analysis and IFA. For Western blots, Vero cells were inoculated with PRV SA215/VP2 or PRV SA215 for 72 h in 6 cm×10 cm cell culture flasks. Twenty microliters of each of the virus-infected cell lysates were separated by SDS-polyacrylamide gel electrophoresis (SDS-PAGE) followed by transfer onto nitrocellulose membranes as previously described [13]. Western blot was carried out using polyclonal anti-VP2 rabbit antibody (1:200 dilution) and HRP-conjugated goat anti-rabbit IgG (1:5000 dilution, Southern Biotechnology, USA) as the primary and secondary antibodies respectively.

For IFA, Vero cells were seeded onto a slide in a six-well plate and then inoculated with PRV SA215/VP2 or PRV SA215 individually. After 24 h inoculation, the cells were harvested for indirect immunofluorescence assay as previously described [13]. Polyclonal anti-VP2 rabbit antibody (1:200 dilution) and FITC conjugated anti-rabbit IgG (1:100 dilution, Pierce, USA) were used as the primary and secondary antibodies respectively. Fluorescent foci were detected by fluorescence microscopy.

### Electron microscopy analysis

PRV SA215/VP2 and PRV SA215 were grown separately in Vero cells in 6 cm × 10 cm cell culture flasks until obvious CPE were visible. Following digestion with trypsin, Vero cells were harvested and ultrathin section samples were prepared for analysis using electron microscopy. Samples were examined using a Hitachi H-600A transmission electron microscope (Hitachi, Japan).

### Animal immunization and challenge

Twenty eight-day-old piglets (n = 20) were obtained locally. These animals had not received PRV and PPV vaccination and tested negative for PRV and PPV antibodies. The piglets were divided into four groups (n = 5 per group). Group 1 was injected intramuscularly (i.m.) with 5×10^5 ^TCID_50 _of recombinant PRV SA215/VP2. Group 2 was injected i.m. with one standard dose of vector virus PRV SA215. Group 3 was injected i.m. with 2 ml inactivated PPV vaccine (China Animal Husbandry Industry Company Ltd., China). Booster injection was given with the same dose at 14 days post-vaccination. Group 4 was inoculated with 2 ml PBS and served as negative control. Animals were bled at 0, 14, 28, 42 and 56 d after immunization and sera were collected for immunoassays. Animals were challenged with 1×10^6 ^TCID_50 _PRV Fa strain at day 56 d post-vaccination. Clinical symptoms and survival were recorded until 14d post-challenge.

Six 6-months-old primiparous gilts (negative for PRV and PPV antibodies) were divided into two groups (n = 3 per group). Group 1 was injected i.m. at 0 d with 5×10^5 ^TCID_50 _PRV SA215/VP2. Group 2 was not immunized and served as negative control. All gilts were challenged at 77 d with 1×10^6.5 ^TCID_50 _PPV SC1 strain via the ear vein. Sera were collected at 0, 28, 77, 114 d. Mummified, stillborn and live foetuses were counted after delivery. Tissue samples from lung, liver, spleen and kidney were obtained from all foetuses, pooled and analyzed for the presence of PPV by PCR.

### Serum neutralization test for PRV

Sera were inactivated at 56°C for 30 min and then diluted two-fold in DMEM in a 96-well flat-bottomed tissue culture plates (Nunc, USA). Virus suspension with a titer of 100 TCID_50 _in 50 μl was added to each serum sample and then incubated for 1 h at 37°C and 5% CO_2_. Vero cell suspension (50 μl) was added to each well and incubated for 3 to 5 days. Appropriate serum, virus and cell controls were included in this test. The plates were monitored for CPE by light microscopy.

### Antibodies detection of PPV

Anti-PPV antibody titers were detected by indirect ELISA using a commercial kit (The GreenSpring TM Porcine Parvovirus ELISA Test Kit, Shenzhen Lvshiyuan Biotechnology Co., Ltd, China) according to the instructions provided by the manufacturer.

### Statistical analysis

An analysis of variance (ANOVA) and a Student's t-test were used to evaluate differences in humoral immune responses between groups. P-values of < 0.05 were considered statistically significant.

## Results

### Construction and identification of recombinant pseudorabies virus PRV SA215/VP2

**Figure 2:**
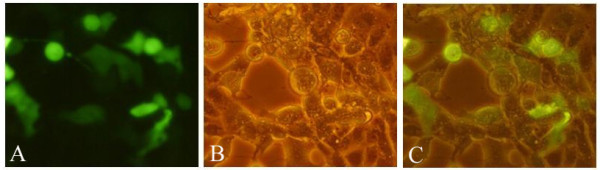
**Expression of the fused EGFP-VP2 protein in Vero cell foci detected by fluorescence microscopy (magnification 400 ×)**. (A) Fluorescence microscope image of PRV SA215/VP2 infected Vero cells 36 h post-infection; (B) Light microscope image of PRV SA215/VP2 infected Vero cells 36 h post-infection; (C) Merged images of (A) and (B).

**Figure 3:**
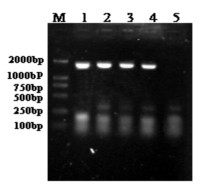
**Identification of recombinant PRV SA215/VP2 by PCR amplication of VP2 gene**. PCR products were analyzed by 0.8% gel agarose electrophoresis. Lanes: (M) Marker DL2000; (1-3) Samples of Vero cells infected with PRV SA215/VP2; (4) Positive control, PPV; (5) Vero cells infected with vector virus PRV SA215.

Recombinant PRV SA215/VP2 was generated through homologous recombination of transfer plasmid pPI-2.EGFP.VP2 and genomic DNA of vector virus PRV SA215 (Figure 1). Obvious CPE were observed approximately three days after co-transfection when PRV SA215/VP2 was harvested. Recombinant viruses were purified for five times by plaque-formation assay. Infection Vero cells showed green fluorescence by fluorescent microscopy (Figure 2). The recombinant virus was identified by PCR amplification of the VP2 gene (Figure 3). The growth rate and viral yields of recombinant viruses were similar to those of the vector PRV SA215. These results confirmed that insertion of the foreign gene did not affect the normal replication of PRV.

### Expression of VP2 gene in recombinant PRV SA215/VP2

**Figure 4:**
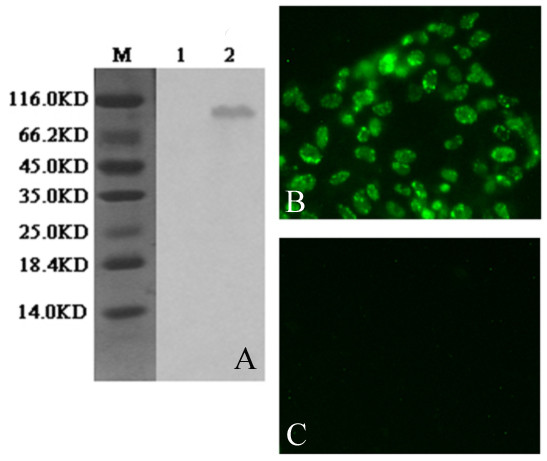
**Analysis of VP2 gene expression**. (A) Western blot analysis of VP2 protein expressed by infected Vero cells. Lanes: (M) Pre-stained protein markers, (1) Vector PRV SA215; (2) Recombinant PRV SA215/VP2. The 93-kDa band represents EGFP and VP2 fusion protein. (B and C) Indirect immunofluorescence analysis of VP2 protein expressed in Vero cells infected with recombinant PRV SA215/VP2 (B) and vector PRV SA215 (C). Cells infected with recombinant PRV SA215/VP2 developed immunofluorescence (B), and cells infected with vector virus PRV SA215, did not develop immunofluorescence (C).

To analyze expression of the VP2 protein, Vero cells were infected with the recombinant PRV SA215/VP2 or vector PRV SA215. Cell proteins were analyzed by Western blot and indirect immunofluorescence. A specific 93-kDa band correlating with the EGFP-VP2 fusion protein was detected in PRV SA215/VP2 infected cells by Western blot analysis, but not in the vector PRV SA215 infected control (Figure 4A). Moreover, in the IFA assay, fluorescence staining was observed in Vero cells infected PRV SA215/VP2 (Figure 4B), but not in the PRV SA215 infected control (Figure 4C).

### Electron microscopy analysis

**Figure 5:**
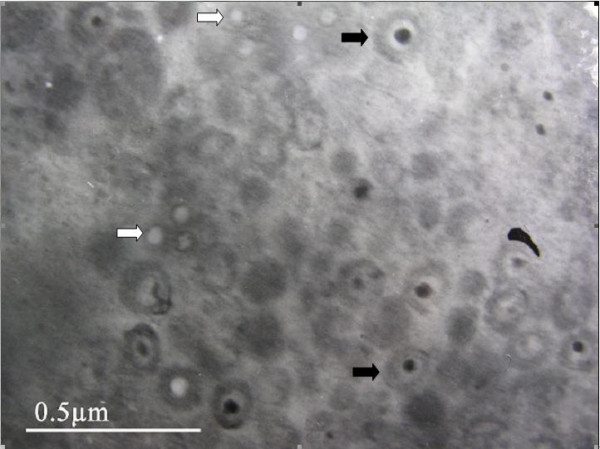
**Electron microscope analysis of Vero cells infected PRV SA215/VP2**. Two types of virus particles were observed in the same Vero cell. Recombinant PRV particles are indicated by the black arrow. Empty virus-like particles probably formed from PPV VP2 protein are indicated by the white arrow.

Electronic microscopy analysis confirmed the presence of two kinds of virus particles in the same Vero cells infected with PRV SA215/VP2. Virus particles of approximately 150 nm exhibited envelope protein and morphological features similar to those of pseudorabies virus. Smaller virus particles of approximately 30-40 nm were identified as empty capsids and exhibited a similar morphology to porcine parvovirus (Figure 5). These results indicate VP2 VLP assembly in the Vero cells infected with recombinant PRV SA215/VP2.

### Neutralizing antibodies against PRV induced by PRV SA215/VP2 and challenge

Neutralizing antibodies against PRV were detected 14 days post-immunization in the sera of piglets immunized with PRV SA215/VP2 or the PRV SA215 vector. No significant differences were detected in the levels of neutralizing antibodies in piglets immunized with PRV SA215/VP2 compared with those vaccinated with PRV SA215 (P > 0.05) (Table 1). No neutralizing antibodies against PRV were be detected in piglets injected with inactivated PPV vaccine and PBS (Table 1).

**Table 1:** PRV-specific neutralizing antibodies (NA) in piglets (X ± SD, n = 5)

Group	0d^a^	14d	28d	42d	56d
PRV SA215/VP2	- ^b^	18.69 ± 3.19	57.61 ± 11.98	65.73 ± 8.04	67.8 ± 6.95
PRV SA215	-	20.12 ± 2.51	62.05 ± 12.52	66.22 ± 11.03	67 ± 6.63
Inactivated PPV vaccine	-	-	-	-	-
PBS	-	-	-	-	-

Note: ^a^Days after vaccination. ^b ^NA titers < 2

Fifty-six days after immunization, all piglets were challenged with 1×10^6 ^TCID_50 _of the virulent PRV Fa strain. Before challenge, all piglets were clinically healthy. After challenge, piglets immunized with PRV SA215/VP2 or PRV SA215 showed no clinical signs of Aujeszky's disease. However, in the inactivated PPV vaccine group and the PBS control group, some piglets showed neurological symptoms of PRV infection before death (Table 2). These data indicate that the recombinant PRV SA215/VP2 provided complete protection against a lethal PRV challenge.

**Table 2:** Piglets immunized with recombinant PRV SA215/VP2 survival following challenge with a lethal dose of virulent PRV Fa strain

Group	Vaccination dose	Challenge dose	Number of piglets	Number of death	Protection (%)
PRV SA215/VP2	5×10^5 ^TCID_50_	1×10^6 ^TCID_50_	5	0	100
PRV SA215	5×10^5 ^TCID_50_	1×10^6 ^TCID_50_	5	0	100
Inactivated PPV vaccine	2ml	1×10^6 ^TCID_50_	5	4	20
PBS	2 ml	1×10^6 ^TCID_50_	5	2	40

### Induction of PPV specific antibodies by PRV SA215/VP2

**Figure 6:**
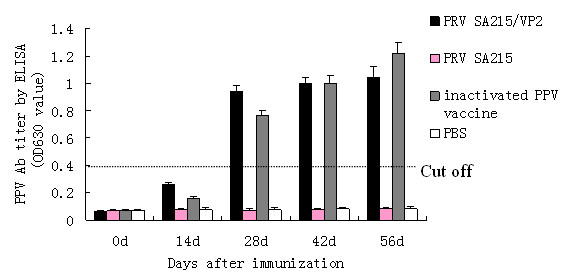
**Immunogenicity of PRV SA215/VP2 in piglets**. Four groups of piglets (n = 5 per group) were immunized with recombinant PRV SA215/VP2, vector PRV SA215, inactivated PPV vaccine and PBS. Data represent the mean antibody titer of each group of piglets determined by indirect ELISA at 0, 14, 28, 42 and 56 d. The horizontal line represents cut-off value.

PPV specific antibodies in sera of piglets were determined using an indirect ELISA assay. Two weeks after vaccination, antibodies against PPV were detected in the sera of piglets immunized with PRV SA215/VP2 and inactivated PPV. The levels of antibodies increased from 14 d to 56 d post-immunization. The antibody titer in the recombinant PRV SA215/VP2 group was significantly higher than that in the inactivated PPV vaccine group at 14 d and 28 d post-immunization (P < 0.01) and significantly lower at 56 d (P < 0.05). There were no significant differences between these two groups at 42 d after immunization. In contrast, no PPV specific antibodies were detected in the PRV SA215 immunized group and PBS control group (Figure 6). These results demonstrate that a PPV-specific antibody response was induced in animals immunized with the recombinant virus.

### Induction of PPV specific antibodies in gilts by PRV SA215/VP2 and viral challenge

**Figure 7:**
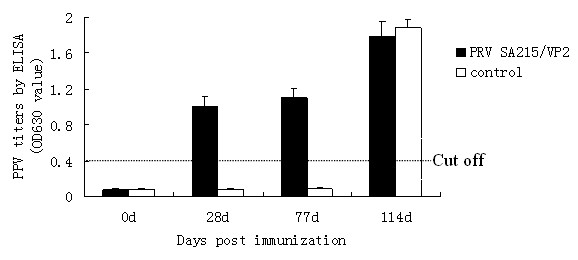
**Immunogenicity of PRV SA215/VP2 in gilts**. Six gilts were divided into two groups. Group 1 was immunized with recombinant PRV SA215/VP2, and group 2 served as negative control. Data represent the mean antibody titer of each group of gilts determined by indirect ELISA at 0, 28, 77 and 114 d. The horizontal line represents cut-off value.

All primiparous gilts remained clinically healthy throughout the experiment. A positive PPV specific antibody response was detected at 28 d in gilts immunized with recombinant PRV SA215/VP2. The level of specific antibodies was maintained up to 77 d. PPV specific antibodies were not detected in the non-immunized group. The specific antibody titer increased in all animals following viral challenge at 77 d (Figure 7). Mortality rates of foetuses of the immunized and non-immunized gilts were 3.6% (1/28) and 22.6% (7/31), respectively. PPV virus DNA was detected in all control group foetuses by PCR, but in none of the recombinant PRV SA215/VP2 immunized group (Table 3). These results indicate that the foetuses of gilts vaccinated with PRV SA215/VP2 were not infected following PPV challenge.

**Table 3:** Number of live and stillborn foetuses and virological analysis of fetal tissues following PPV challenge.

Group	Total fetuses	Live foetuses	Stillborn foetuses	PPV-PCR positive No (%)
PRV SA215/VP2	28	27	1	0(0)
Control	31	24	7	31(100)

## Discussion

Vaccines play a critical role in the control of viral diseases. Vaccines against both PRV and PPV are extensively used all over the world. However, the use of such vaccines in clinical practice is limited by considerations of time, cost, safety and poor PPV replication in vitro. Therefore, research is now focused on developing a bivalent vaccine.

The parent PRV strain SA215 used in this trial is an approved vaccine strain which is strongly immunogenic and has been widely used to prevent and control pseudorabies diseases in China. This PRV vaccine strain has been attenuated by deletion of the genes encoding three important virulence factors (TK, gE, and gI) of the Fa strain. It has been approved as a new veterinary medicine and is the first animal virus-based genetically engineered vaccine in China.

Many systems have been successfully used for the expression of the PPV VP2 gene resulting in self-assembly of VLPs. Such systems include adenovirus, yeast, and particularly, the insect cell-baculovirus system [7,21,22] which has been used for mass production. Their expression products assembled into VLPs were similar in size and morphology to the original virions. The highly immunogenic VLPs have been shown to protect breeding sows against reproductive failure following virulent virus challenge [23], which is in accordance with the data obtained in this study. However, the potential for contamination of PPV VLPs with the vectors used in these approaches has raised serious safety concerns about the use of these recombinant vaccines [24,25]. In this study, the pseudorabies virus system, which was extensively applied to express other heterologous antigens, was used to express the PPV VP2 gene successfully, without any of the described safety issues.

Recently, PPV VLPs have been shown to express foreign polypeptides in certain positions, resulting in the successful production of many highly immunogenic peptides, and the induction of strong antibody, helper-T-cell, and cytotoxic T-lymphocyte responses [26]. Sedlik et al. prepared the hybrid virus-like particles by self-assembly of the modified porcine parvovirus VP2 capsid protein, carrying a CD8+ T cell epitope from the lymphocytic choriomeningitis virus nucleoprotein. Immunization of mice with these hybrid pseudoparticles, without any additional adjuvant, induced strong cytotoxic T lymphocyte (CTL) responses against both peptide-coated and virus-infected target cells [27]. Pan et al. used PPV VLPs as a new generation of non-replicative vectors for delivery of a PCV2 epitope, which was expressed in adenovirus. The expressed product significantly enhanced both antibody-specific and cell-mediated immune response to PPV and PCV2 [28]. It can be speculated that the PRV SA215/VP2 constructed in this study could be used for the insertion of foreign polypeptides in specific positions of the VP2 gene to enhance immunogenicity of PRV and PPV, and furthermore, may be developed as a multivalent vaccine.

The VP2 and EGFP fusion protein facilitated screening and purification of the recombinant virus from infected Vero cells although the issue of correct folding of the VP2 protein was subsequently addressed. An independent VP2 expression cassette was constructed which induced a higher level of antibody production. Positive identification of VP2 protein by Western blot analysis and IFA assay using a polyclonal anti-VP2 rabbit antibody indicated correct folding of VP2 protein, thus, providing the essential component for vaccine development. Safety of the recombinant virus was indicated by the absence of clinical signs related to Aujeszky's disease in piglets and gilts immunized with recombinant virus. Furthermore, no difference in virulence was detected between the recombinant virus and the vector virus. Efficacy experiments demonstrated that piglets immunized with the recombinant virus elicited PRV-specific and PPV-specific humoral immune responses and provided complete protection against a lethal PRV challenge, which was in agreement with other reports [8,10,29]. Furthermore, gilts immunized with the recombinant virus induced PPV-specific antibodies and provided complete protection against challenge with the PPV-SC1 strain. These results demonstrated the immunogenicity of the recombinant virus.

Moreover, this genetically engineered vaccine also allowed differentiation between vaccinated and the naturally infected pigs, thus contributing to the Aujeszky's disease eradication program.

## Conclusions

A recombinant virus PRV SA215/VP2 expressing the PPV VP2 protein was constructed using the PRV SA215 as a vector. This study demonstrated the safety, immunogenicity, and protective efficacy of this vaccine in piglets and primiparous gilts. This recombinant virus represents a suitable candidate for further clinical evaluation of its application as a bivalent vaccine against both PRV and PPV infection.

## Competing interests

The authors declare that they have no competing interests.

## Authors' contributions

YC, YL, QS carried out most of the experiments and drafted the manuscript. WZG and ZWX critically revised the manuscript and the experiment design. DSC, LZ, XYW helped with the experiment. All of the authors read and approved the final version of the manuscript.

## References

[B1] Krakowka S Ellis JA Meehan B Kennedy S McNeilly F Allan G Viral wasting syndrome of swine: experimental reproduction of postweaning multisystemic wasting syndrome in gnotobiotic swine by co infection with porcine circovirus 2 and porcine parvovirus Vet Pathol 2000 37 3 254 63 10.1354/vp.37-3-254 10810990

[B2] Kresse JI Taylor WD Stewart WW Eernisse KA Parvovirus infection in pigs with necrotic and vesicle-like lesions Veterinary Microbiology 1985 10 6 525 531 10.1016/0378-1135(85)90061-6 3006323

[B3] Allan GM Kennedy S McNeilly F Foster JC Ellis JA Krakowka SJ Meehan BM Adair BM Experimental Reproduction of Severe Wasting Disease by Co-infection of Pigs with Porcine Circovirus and Porcine Parvovirus Journal of Comparative Pathology 1999 121 1 1 11 10.1053/jcpa.1998.0295 10373289

[B4] Paul PS Mengeling WL Brown TT Effect of vaccinal and passive immunity on experimental infection of pigs with porcine parvovirus Am J Vet Res 1980 41 9 1368 71 7447129

[B5] Mengeling WL Lager KM Vorwald AC The effect of porcine parvovirus and porcine reproductive and respiratory syndrome virus on porcine reproductive performance Anim Reprod Sci 2000 60-61 199 210 10.1016/S0378-4320(00)00135-4 10844195

[B6] Mengeling WL Brown TT Paul PS Gutekunst DE Efficacy of an inactivated virus vaccine for prevention of porcine parvovirus-induced reproductive failure Am J Vet Res 1979 40 2 204 7 464358

[B7] Martinez C Dalsgaard K Lopez de Turiso JA Cortes E Vela C Casal JI Production of porcine parvovirus empty capsids with high immunogenic activity Vaccine 1992 10 10 684 90 10.1016/0264-410X(92)90090-7 1523879

[B8] Yuan Z Zhang S Liu Y Zhang F Fooks AR Li Q Hu R A recombinant pseudorabies virus expressing rabies virus glycoprotein: safety and immunogenicity in dogs Vaccine 2008 26 10 1314 21 10.1016/j.vaccine.2007.12.050 18262313

[B9] Li X Liu R Tang H Jin M Chen H Qian P Induction of protective immunity in swine by immunization with live attenuated recombinant pseudorabies virus expressing the capsid precursor encoding regions of foot-and-mouth disease virus Vaccine 2008 26 22 2714 22 10.1016/j.vaccine.2008.03.020 18436351

[B10] Jiang Y Fang L Xiao S Zhang H Pan Y Luo R Li B Chen H Immunogenicity and protective efficacy of recombinant pseudorabies virus expressing the two major membrane-associated proteins of porcine reproductive and respiratory syndrome virus Vaccine 2007 25 3 547 60 10.1016/j.vaccine.2006.07.032 16920232

[B11] Lin Y Qigai H Xiaolan Y Weicheng B Huanchun C The co-administrating of recombinant porcine IL-2 could enhance protective immune responses to PRV inactivated vaccine in pigs Vaccine 2005 23 35 4436 41 10.1016/j.vaccine.2005.03.034 15946776

[B12] Xu G Xu X Li Z He Q Wu B Sun S Chen H Construction of recombinant pseudorabies virus expressing NS1 protein of Japanese encephalitis (SA14-14-2) virus and its safety and immunogenicity Vaccine 2004 22 15-16 1846 53 10.1016/j.vaccine.2003.09.015 15121294

[B13] Song Y Jin M Zhang S Xu X Xiao S Cao S Chen H Generation and immunogenicity of a recombinant pseudorabies virus expressing cap protein of porcine circovirus type 2 Vet Microbiol 2007 119 2-4 97 104 10.1016/j.vetmic.2006.08.026 17005335

[B14] Yin J Ren X Tian Z Li Y Assembly of pseudorabies virus genome-based transfer vehicle carrying major antigen sites of S gene of transmissible gastroenteritis virus: potential perspective for developing live vector vaccines Biologicals 2007 35 1 55 61 10.1016/j.biologicals.2006.02.001 16731004 PMC7128284

[B15] van Zijl M Wensvoort G de Kluyver E Hulst M van der Gulden H Gielkens A Berns A Moormann R Live attenuated pseudorabies virus expressing envelope glycoprotein E1 of hog cholera virus protects swine against both pseudorabies and hog cholera J Virol 1991 65 5 2761 5 1850051 10.1128/jvi.65.5.2761-2765.1991 PMC240645

[B16] Klupp BG Hengartner CJ Mettenleiter TC Enquist LW Complete, annotated sequence of the pseudorabies virus genome J Virol 2004 78 1 424 40 10.1128/JVI.78.1.424-440.2004 14671123 PMC303424

[B17] Olsen LM Ch'ng TH Card JP Enquist LW Role of pseudorabies virus Us3 protein kinase during neuronal infection J Virol 2006 80 13 6387 98 10.1128/JVI.00352-06 16775327 PMC1488934

[B18] Kimman TG de Wind N Oei-Lie N Pol JM Berns AJ Gielkens AL Contribution of single genes within the unique short region of Aujeszky's disease virus (suid herpesvirus type 1) to virulence, pathogenesis and immunogenicity J Gen Virol 1992 73 Pt 2 243 51 1311354 10.1099/0022-1317-73-2-243

[B19] Chen L Guo W Xu Z Wang X Wang Y The Biological Characteristics of a Gene-deleted Pseudorabies Virus Vaccine Strain (SA215) Chinese Journal of Animal and Veterinary Sciences 2005 36 3 278 282

[B20] Guo W Xu Z Wang X Chen L Wang Y Wang M Construction of new generation Pseudorabies virus gene deleted vaccine and the study of biologic characteristics Journal of Sichuan Agricultural University 2000 18 1 1 10

[B21] Casal JI Rueda P Hurtado A Use of parvovirus-like particles for vaccination and induction of multiple immune responses Biotechnol Appl Biochem 1999 29 Pt 2 141 50 10075910

[B22] Zhou H Yao G Cui S Production and purification of VP2 protein of porcine parvovirus expressed in an insect-baculovirus cell system Virol J 2010 7 366 10.1186/1743-422X-7-366 21143963 PMC3022681

[B23] Casal JI Parvovirus diagnostics and vaccine production in insect cells Cytotechnology 1996 20 1 261 270 10.1007/BF00350405 22358489

[B24] Antonis AFG Bruschke CJM Rueda P Maranga L Casal JI Vela C Hilgers Luuk AT Belt Peter BGM Weerdmeester K Carrondo Manuel JT Langeveld Jan PM A novel recombinant virus-like particle vaccine for prevention of porcine parvovirus-induced reproductive failure Vaccine 2006 24 26 5481 5490 10.1016/j.vaccine.2006.03.089 16730104

[B25] Maranga L Cunha A Clemente J Cruz P Carrondo MJ Scale-up of virus-like particles production: effects of sparging, agitation and bioreactor scale on cell growth, infection kinetics and productivity J Biotechnol 2004 107 1 55 64 10.1016/j.jbiotec.2003.09.012 14687971

[B26] Casal JI Rueda P Hurtado A Parvovirus-Like Particles as Vaccine Vectors Methods 1999 19 1 174 186 10.1006/meth.1999.0843 10525454

[B27] Sedlik C Saron M Sarraseca J Casal JI Leclerc C Recombinant parvovirus-like particles as an antigen carrier: a novel nonreplicative exogenous antigen to elicit protective antiviral cytotoxic T cells Proc Natl Acad Sci USA 1997 94 14 7503 8 10.1073/pnas.94.14.7503 9207121 PMC23851

[B28] Pan Q He K Huang K Development of recombinant porcine parvovirus-like particles as an antigen carrier formed by the hybrid VP2 protein carrying immunoreactive epitope of porcine circovirus type 2 Vaccine 2008 26 17 2119 2126 18378364 10.1016/j.vaccine.2008.02.037

[B29] Zuckermann FA Aujeszky's disease virus: opportunities and challenges Vet Res 2000 31 1 121 31 10726641 10.1051/vetres:2000111

